# Beyond money: Risk preferences across both economic and non-economic contexts predict financial decisions

**DOI:** 10.1371/journal.pone.0279125

**Published:** 2022-12-16

**Authors:** Crystal Reeck, O’Dhaniel A. Mullette-Gillman, R. Edward McLaurin, Scott A. Huettel

**Affiliations:** 1 Department of Marketing, Fox School of Business, Temple University, Philadelphia, Pennsylvania, United States of America; 2 Center for Cognitive Neuroscience, Duke University, Durham, North Carolina, United States of America; 3 Department of Psychology & Neuroscience, Duke University, Durham, North Carolina, United States of America; Universidad de Alicante, SPAIN

## Abstract

Important decisions about risk occur in wide-ranging contexts, from investing to healthcare. While an underlying, domain-general risk attitude has been identified across contexts, it remains unclear what role it plays in shaping behavior relative to more domain-specific risk attitudes. Clarifying the relationship between domain-general and domain-specific risk attitudes would inform decision-making theories and the construction of decision aids. The present research assessed the relative contribution of domain-general and domain-specific risk attitudes to financial risk taking. We examined risk attitudes across different decision domains, as revealed through a well-validated measure, the Domain-Specific Risk-Taking Scale (DOSPERT). Confirmatory factor analysis indicated that a domain-general risk attitude shaped responses across multiple domains, and structural equation modeling showed that this domain-general risk attitude predicted observed behavioral risk premiums in a financial decision-making task better than domain-specific financial risk attitudes. Thus, assessments of risk attitudes that include both economic and non-economic domains improve predictions of financial risk taking due to the enhanced insight they provide into underlying, domain-general risk preferences.

## Introduction

High-stakes decisions involving risk occur across a wide range of contexts, from medical treatments to financial investments. Across such varying domains, are decisions shaped by domain-specific attitudes or by a domain-general attitude towards risk? Financial advisors suggest investments by assessing their clients’ risk attitudes, yet determining which risk attitudes are relevant can prove challenging. Suppose that a retirement saver enjoys skydiving; should she be guided to riskier assets or is her recreational hobby irrelevant to her financial preferences? While earlier research emphasized that risk attitudes vary across different domains [1,2], more recent research indicates there is also an underlying, domain-general attitude that shapes choices across domains [3–5]. However, it remains unclear how this domain-general risk attitude relates to economic risk taking. The present research assessed the relative contribution of domain-general and domain-specific risk attitudes to financial risk taking.

Risk-taking behavior differs across contexts (e.g., [6]). For example, investors respond to risk differently if they are investing company or personal money [7], doctors make different choices for themselves than their patients [8], recent successes lead people to accept additional risk [9], and people opt for different strategies when choosing between potential products or potential spouses [10]. This observation that risk-taking varies across contexts challenges traditional economics theories of risk preferences, often based on expected utility theory, that typically assert risk preferences are stable and unidimensional (e.g., [11]). It also contrasts with work in psychology that classifies individuals as impulsive or not without considering context-specific variability (e.g., [12]). Responding to the need to measure variability in risk attitudes across contexts, the Domain-specific Risk-Taking Scale (DOSPERT) has revealed several domains of risk-taking: financial, ethical, recreational, health and safety, and social [1,2]. Self-reported risk attitudes on the DOSPERT track actual risk-taking behaviors in different domains [2,13], demonstrating the predictive validity of assessing domain-specific risk attitudes.

Although context clearly modulates risk attitudes and risk-taking behavior, those observations do not preclude the existence of another, domain-general component, such that an individual’s decisions are the result of preferences that are relatively stable across domains (e.g., [11,14]). Some empirical evidence supports the existence of such domain-general preferences. Although individuals perceive varying amounts of risk in different contexts, their overall attitude towards perceived risk is relatively stable [2,9], and decisions about risk are correlated across different domains [2,4,15]. Recent examinations demonstrate that there is a domain-general component to risk preferences more generally [4] and to responses on the DOSPERT specifically [3,5]. Indeed, a domain-general component to risk preferences has been identified across a broad range of measures with good test-retest reliability, suggesting domain-general risk preferences share features with other stable psychological traits [4]. However, while there is evidence for a domain-general risk factor, the relative contribution of domain-general and domain-specific risk attitudes to shaping economic risk taking remains unclear.

Counterintuitively, the existence of a domain-general component to risk attitudes suggests that the predictive power within any one domain would be enhanced with the inclusion of preferences from other domains. As many preferences are constructed [16], an improved estimate of one’s general propensity toward risk may play a key role in shaping behavior, perhaps explaining choice better than attitudes from more proximal domains. For example, consider the sky-diver weighing her options for retirement savings–even though she may pursue risk recreationally by sky-diving, she may actually view the activity as relatively safe, given her past pleasant experiences and familiarity with safety checks in place. In this case, her perceived risk would be low. Looking across domains at how she approaches other risks would elucidate her more general propensity toward risk, informing how she will approach new decisions presented to her. Indeed, domain-general risk attitudes may subsume domain-specific attitudes at predicting actual risk taking.

The present research builds on several previous empirical demonstrations. For example, Dohmen and colleagues asked survey respondents to self-report their willingness to take risks [17]. Self-reported willingness to take risks tracked several relevant risky behaviors, including a decision about a lottery and whether their household invested in stocks. Frey and colleagues examined the factor structure of the DOSPERT in a large sample of respondents, finding evidence for both domain-general and domain-specific components of risk attitudes on this measure [3]. Highhouse and colleagues similarly found evidence for both domain-general and domain-specific components to risk attitudes using the DOSPERT [5]. They also examined the relative contributions of these components to potentially relevant behaviors, such as self-reported counterproductivity and workplace safety. However, while this previous body of work provides compelling evidence for a domain-general component to risk attitudes, the relative influence of domain-general and domain-specific risk attitudes on financial risk taking remains unclear.

Clarifying separable domain-specific and domain-general risk and their relative contribution to behavior is fundamental to informing theories of decision making. Identifying domain-general processes and abilities has been fruitful in multiple areas of psychology, including intelligence [18], working memory [19], and critical thinking [20]. The present experiment evaluates the relative contributions of domain-general and domain-specific risk attitudes in predicting incentive-compatible financial risk taking, with the aim of informing basic decision-making theories and behavioral interventions to support consumer decisions under uncertainty.

We find evidence for both domain-general and domain-specific components of risk attitudes, but demonstrate that the former rather than the latter predominantly track financial behavioral risk premiums. Our results make three key contributions to understanding the psychology of risk. First, we replicate and extend prior empirical demonstrations that there is a domain-general component to risk attitudes [3–5]. Importantly, in contrast to these prior demonstrations that relied on bifactor models, we adopt a second-order model, which is the more common and parsimonious approach [21]. The consistency of our findings with prior demonstrations highlights the robustness of the finding of domain-general risk attitudes across analytical approaches. Second, we show that domain-general components of risk attitudes track financial behavioral risk premiums better than domain-specific components using an incentive-compatible economic decision-making task. Whereas prior research examining the relative influence of domain-general and domain-specific risk attitudes on behavior has predominantly relied on self report, our approach enables us to more precisely and validly measure actual financial risk taking. Finally, the present results help resolve theoretical debate regarding the nature of risk preferences. Traditional approaches in both economics and psychology have often conceptualized risk attitudes as stable across contexts (e.g., [11,12]); other approaches have emphasized the variability in risk attitudes across contexts (e.g., [1,2,13]). The present results help unify these two frameworks by demonstrating that risk attitudes are simultaneously domain-general and domain-specific, consistent with the hypothesis that people have differing perceptions of risks in specific contexts but a fundamental predisposition towards risk when it is perceived [2,9]. These findings can help inform the design of decision aids, assessments, and behavioral interventions to promote consumer financial welfare. For example, these insights could be leveraged to develop new techniques for soliciting consumers’ risk preferences in order to ensure their choices in a less familiar domain align with their risk attitudes in other domains. Alternately, these insights could also inform policies or choice architecture interventions seeking to promote consumer welfare by encouraging the adoption of beneficial options [22].

## Methods

### Participants

Our sample comprised three hundred and four adult participants (169 women; *M* = 27.1 years, *s*.*d*. = 10.4) recruited for in-person sessions from the Duke University and Durham, North Carolina, community. The participant sample was ethnically heterogeneous (65.8% White, 14.8% Asian, 7.9% Black, 2.0% Hispanic, 1.6% Middle Eastern or Central Asian, 0.3% Pacific Islander, 7.2% Multi-ethnic, 0.3% Decline to state). All participants provided written informed consent under a protocol approved by the Duke University Medical Center Institutional Review Board and the session lasted approximately one hour. On average, participants earned approximately $23.90 during the experiment (range $15 - $56), reflecting a combination of both hourly compensation and incentives based on their choices in the experiment (see below).

### Materials & procedure

Participants completed the DOSPERT [2] to gauge self-reported risk attitudes across a variety of domains (see [2], Appendix C for specific items). The DOSPERT contains forty questions targeting six separate domains of risk (i.e., Investing, Gambling, Health/Safety, Ethical, Social, and Recreational). We focused on its risk-behavior subscale, in which participants indicate their likelihood of engaging in the behavior described in each item using a five-point Likert scale ranging from “Extremely Unlikely” to “Extremely Likely.” No participants were missing responses to any items on the DOSPERT.

After completing the DOSPERT, participants also completed 120 trials of an incentive-compatible risk preference task [23,24]. On each trial (**S1 Fig**), participants chose between two options: a certain payout (e.g., a guaranteed payment of $5) and a risky payout (e.g., a gamble with a 50% probability of $16 and a 50% probability of $0). Unique options were presented on each trial of the task and the trial order was randomly determined for each participant. The side on which each option was presented was counterbalanced across trials, to avoid either option becoming associated with a response key. The set of 120 unique options was generated by crossing five potential values for the certain outcome ($3, $4, $5, $6, $7) with three possible probabilities of winning the gamble option (25%, 50%, 75%) and eight potential ratios between the expected value of the gamble option and the value of the certain option (0.5, 1.0, 1.3, 1.6, 1.9, 2.2, 2.5, 3.0). The risky option always consisted of two possible outcomes: one positive and one $0. This design feature minimized the influence of loss aversion on decisions. Sample participant instructions are included in the S1 Supplemental materials. Information regarding the outcomes of the risky options was not presented to the participant to prevent the outcomes of decisions from altering choices on subsequent trials. At the end of the experiment, one trial was randomly selected and the participant’s decision played out to determine their bonus for task participation. Thus, the participant was incentivized to respond in accordance with their genuine preferences. As part of a larger study of individual differences in risk and reward processing, participants provided additional self-report, behavioral, and biological data not considered here. Data are available through the Open Science Framework (https://osf.io/wrc3e/).

### Data analysis

Data from the decision-making task were analyzed in MATLAB (Mathworks, Inc.). Each participant’s risk indifference point was computed from their choices to determine the relative ratio of expected values necessary to equate the attractiveness of the risky gamble and the certain option. For each person, we examined the percentage of times they selected the risky gamble instead of the certain option based on the ratio between the expected values for each option. This relationship is typically monotonic, and indifference points were calculated based on the expected value ratio at which participants’ likelihood of selecting the risky option was approximately 50%. For gambles in the gain domain, like those used in the present study, individuals tend to be risk-averse [6], which was reflected in high relative expected values necessary for the risky option to be as attractive as the certain option. Indifference points could not be calculated for forty-one participants in the present experiment, as their choices never indicated they had crossed their indifference point (for instance, some participants always selected the certain option). Calculated indifference points were transformed to risk premium values to provide a zero-centered scale (with zero as risk neutral, positive for risk aversion, and negative for risk seeking) and to specify the additional relative expected value necessary to produce indifference between the certain option and the risky option [24]. The risk premiums reflect the percentage of additional expected value of the risky option necessary for participants to be indifferent between the risky option and the certain option.

We utilized structural equation modeling to examine the responses to the DOSPERT and their relationship with risk premiums. Compared to other statistical approaches that focus on estimating means (e.g., ANOVA), structural equation modeling focuses on modeling the covariance between variables. This approach involves estimating the latent variables guiding observed responses (e.g., responses to individual survey questions) and typically has two components: the measurement model and the structural model. Following common practice, we employed confirmatory factor analysis for the measurement model. This phase enabled us to model the latent variables shaping responses on the DOSPERT. Specifically, we focused on examining whether responses were solely guided by domain-specific components or were also influenced by a domain-general component. Following acceptable fit of the measurement model, the structural model examined the relationship between financial behavioral risk premiums and domain-specific and domain-general risk attitudes. The confirmatory factor analysis and structural equation modeling were conducted using MPlus Version 6.1 [25].

In the measurement model, DOSPERT responses from the risk-behavior scale were treated as categorical indicators and models were estimated employing the weighted least squares mean and variance adjusted (WLSMV) estimator (which is commonly used when data are categorical and potentially non-normal). Means, standard deviations, and correlations for all variables included in the models can be found in **S1 Table**. Confirmatory factor analysis was conducted on the DOSPERT responses to assess the measure’s factor structure using two main models (**Supplemental Methods**). Previous exploratory factor analyses of the measure were used as the basis for confirmatory factor analysis [2], with individual items loading on six domain-specific factors (i.e., Investing, Gambling, Health/Safety, Ethical, Social, and Recreational). Note that this initial model included only domain-specific factors, but no domain-general factor. This conceptualization is consistent with theoretical accounts that emphasize variability in risk attitudes across contexts (e.g., [2,13]). In order to identify variance in risk attitudes shared across all domain-specific factors, a second comparison model was generated including a second-order, domain-general risk factor in addition to the domain-specific factors previously identified by exploratory factor analysis. This model was identical to the previous model with the addition of a second-order factor upon which all six domain-specific factors loaded. This comparison model is consistent with theoretical accounts highlighting a potential domain-general risk attitude (e.g., [3–5,17]). We then compared the model fits to assess whether including a domain-general component to risk attitudes improves model fit.

**Fig 1:**
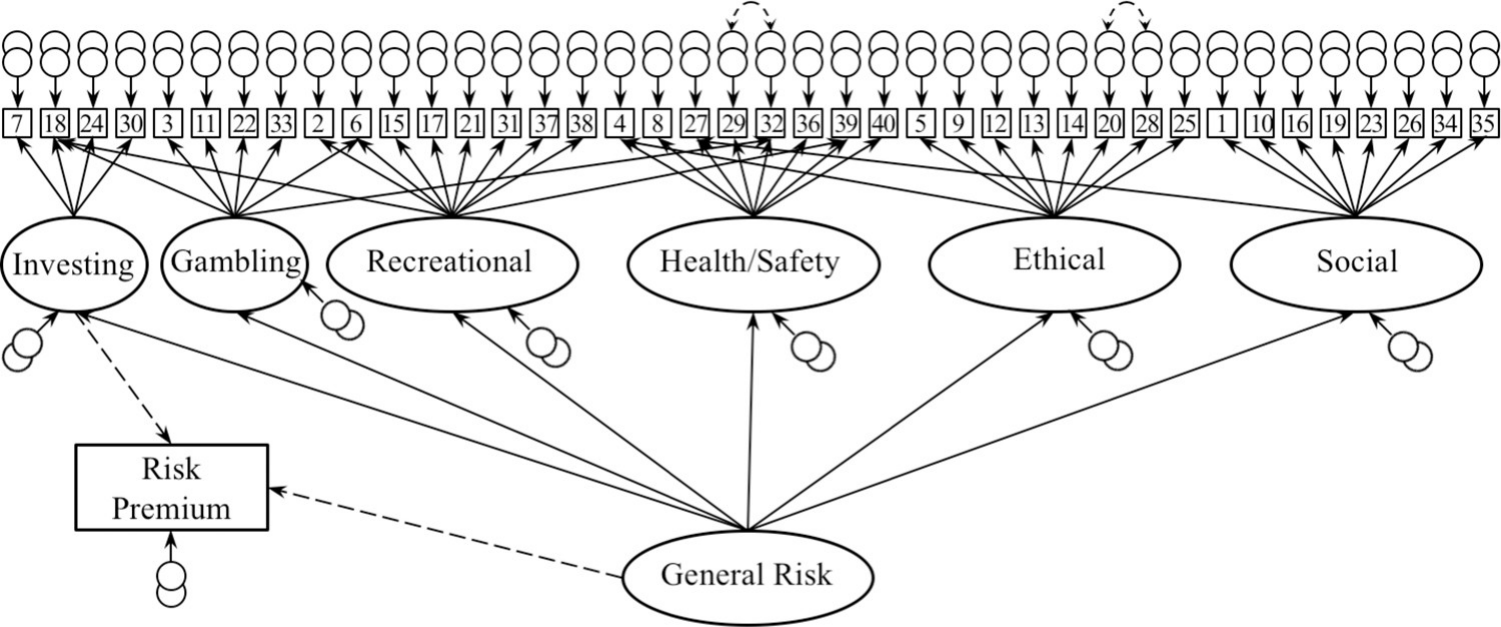
Diagram of model tested. Measurement model based on forty observed responses to the risk-behavior scale of the DOSPERT includes six domain-specific factors and one domain-general factor. Dashed lines indicate paths added for structural model testing on independent measures of risky choice behavior. Following standard practice, rectangles represent observed data and ovals represent latent variables.

Following acceptable fit of the measurement model, structural equation modeling was conducted to examine the relationship between domain-general risk attitudes, risk attitudes specific to the Investing domain, and risk premiums identified from the economic risk preferences task **(Supplemental Methods)**. The structural model employed the DOSPERT measurement model from **Fig 1** with the addition of the observed risk premiums from the risk preference task [24]. The loadings from the Investing domain-specific factor and the domain-general risk factor to the observed risk premium data were estimated (dashed lines, Fig 1) and the paths between risk premium and all other variables were fixed at zero.

## Results

### Domain-general preferences shape risk attitudes across domains

Initial analyses investigated whether a domain-general component to risk attitudes shaped responding to the DOSPERT. Towards that end, we estimated two measurement models: a domain-specific structure identified by previous exploratory factor analysis of the DOSPERT [2] and a separate model structure that included a higher-order, domain-general component. If a domain-general component shapes risk attitudes, the model including this latent variable should provide improved fit compared to the model omitting it. If, on the other hand, a domain-general component does not shape risk attitudes, including this latent variable in the model should not significantly alter model fit (or may even degrade it). The overall fit of the purely domain-specific model was poor (χ^2^(740) = 2625.23, *p* < .001; RMSEA = .092, 90% CI = .088, .095; CFI = .56), as measures of fit were well below conventional thresholds of significance (**Table 1**). Although the model including a domain-general factor also exhibited poor overall fit (χ^2^(734) = 1279.62, *p* < .001; RMSEA = .049, 90% CI = .045, .054; CFI = .87), it provided a marked and significant improvement over the domain-specific model (χ^2^(6) = 1345.61, *p* < .001) (**Table 1**). Thus, a domain-general component appears to shape self-reported risk attitudes across multiple domains of risky behavior.

**Table 1:** Fit indices for models estimated using responses to the risk-behavior scale of the DOSPERT (N = 304).

Model	Description	df	χ ^2^	*p*	RMSEA	RMSEA 90% Confidence Interval	CFI
*Measurement Models*							
1	Purely domain-specific	740	2625.23	< .001	.092	.088—.095	.56
2	Domain-general factor included	734	1279.62	< .001	.049	.045—.054	.87
Difference between Models 1 & 2		6	1345.61	< .001			
3	Modified, without domain-general factor	731	2139.49	< .001	.080	.076—.084	.67
4	Modified, with domain-general factor	725	1077.51	< .001	.040	.035—.045	.92
Difference between Models 3 & 4		6	1061.98	< .001			

*Structural Models*							
1	Model depicted Fig 1	763	1104.53	< .001	.038	.033—.043	.92
2	No path from Investing to risk premium	764	1106.80	< .001	.038	.033—.043	.92
Difference between Models 1 & 2		1	2.27	.132			
3	No path from domain-general risk to risk premium	764	1114.66	< .001	.039	.034—.044	.92
Difference between Models 1 & 3		1	10.13	.001			
4	Employing measurement model from original DOSPERT structure	772	1304.28	< .001	.048	.043—.053	.88
Difference between Models 1 & 4		9	199.75	< .001			

Note: df indicates degrees of freedom of each model. *p* values correspond to χ^2^ statistic. Root mean-square error of approximation and 90% confidence interval provided, along with Comparative Fit Index. Measurement Model 4, incorporating a domain-general factor and model modifications, provided the best fit and was the only model meeting conventional thresholds for fit indices. Structural Model 1 denotes the initial structural model tested. Subsequent structural models were conducted to test features of Structural Model 1.

While these initial models provided empirical support for the notion that there is a domain-general component to risk attitudes, both models exhibited less than satisfactory fit. A Lagrange Multiplier Test was implemented to identify potential improvements to the factor structure (see **Supplemental Methods**). The modified model is displayed in **Fig 1**. As employing these modifications involved deviating from the original proposed factor structure, this measurement model is no longer strictly confirmatory. However, this approach is consistent with recent advances in exploratory structural equation modeling [26, 27], which allow cross-loadings for items to deviate from zero. These changes typically reflect covariation in items that emerges when there are many indicators. Please note that this approach avoids concerns related to overfitting as the key components of the models–the domain-specific and domain-general factors–remain unchanged. After incorporating these modifications, model fit improved dramatically and indicated good fit to the data (χ^2^(725) = 1077.51, *p* < .001; RMSEA = .040, 90% CI = .035, .045; CFI = .92).

The measurement model (**S2 Table**) provides strong evidence in favor of a second-order, domain-general component to risk attitudes. All of the loadings from the domain-specific factors to the second-order latent variable are significant (*p*’s < .01), suggesting a core component tracks risk attitudes across all six domains. Specifically, the loadings of the domain-specific components on the domain-general component range from .204 - .859 and areall significantly different from 0. To confirm the central importance of this second-order variable, we estimated an additional model that eliminated this domain-general, second-order component. If omitting the domain-general component degrades model fit, this degradation would be evidence that the domain-general component shapes responses on the DOSPERT. This model exhibited both poor fit to the data (χ^2^(731) = 2139.49, *p* < .001; RMSEA = .080, 90% CI = .076, .084; CFI = .67) and a marked degradation compared to the model including a domain-general factor (χ^2^(6) = 1061.98, *p* < .001). These findings challenge a pure domain-specific interpretation of responses on the DOSPERT and support the inclusion of a second-order, domain-general aspect of risk attitudes.

### Domain-general risk attitudes predict financial risk-taking behavior

Next, structural equation modeling interrogated the relationships among domain-general risk attitudes, domain-specific Investing risk attitudes, and behavior in the economic risk preference task. Measurements of risk premiums were added to the model via pathways from both the domain-general risk attitude latent variable and the domain-specific Investing latent variable (**Fig 1**). This structural model exhibited close fit to the data (χ^2^(763) = 1104.53, *p* < .001; RMSEA = .038, 90% CI = .033, .043; CFI = .92) (**Table 1**). Inspection of the parameters from the structural model (**S3 Table**) indicates that there was a significant negative relationship between domain-general risk attitudes and risk premiums (β = -0.168, *p* = .018), such that greater expected values were required to entice an individual to gamble instead of selecting a lower-payoff but guaranteed alternative. While domain-general risk attitudes exhibited a significant relationship with risk premiums, the relationship between Investing domain risk attitudes and risk premium was only marginally significant (β = -0.124, *p* = .088).

These findings suggest the primacy of domain-general risk attitudes in shaping financial risk-taking in this task. To further interrogate these effects, we conducted several complementary analyses. First, we assessed the independent contributions to model fit of the Investing factor and the domain-general factor by separately fixing each path to zero and re-estimating the model for comparison. Fixing the path between the domain-specific Investing factor and risk premiums resulted in no significant loss of model fit compared to the original structural model (χ^2^(1) = 2.27, *p* = .132), indicating that the exclusion of that path did not dramatically degrade overall model fit. Conversely, eliminating the path between the domain-general factor and risk premiums resulted in a significant and dramatic reduction in model fit (χ^2^(1) = 10.13, *p* < .001), demonstrating the vital importance of this path in contributing to the model. Second, we examined the possibility that other domain-specific factors (e.g., Gambling, Health/Safety, Recreational, etc.) might also share a significant relationship with risk premiums. A Wald test was conducted to discern whether freeing the paths between other domain-specific factors and risk premiums would enhance model fit, but significant model fit improvement was not achieved by freeing any of the paths. Finally, subsequent analyses fixing the Investing path to risk premiums at zero and freeing individual paths to other domain-specific latent variables also failed to produce improved model fit. Therefore, domain-general risk attitudes exhibit the strongest relationship to observed economic risk premiums, whereas domain-specific factors–including the Investing and Gambling factors–do not make significant independent contributions to financial risk-taking.

## Discussion

Our results demonstrate that domain-general risk attitudes track interindividual variability in incentive-compatible, financial risk taking–explaining more variance than domain-specific effects. These findings were robust across different domain-specific factors, supporting a primary role for domain-general risk attitudes in shaping economic behavior.

The present demonstration of the primacy of domain-general risk attitudes is consistent with previous research showing that risk attitudes in one domain can predict risk taking in another domain [2,15,17]. Weber and colleagues theorize that people vary across domains in the amount of risk they perceive, but are consistent in their attitudes towards perceived risk [2,9]. For example, an individual whose risk attitudes depend on whether they understand the underlying rules of an activity might view gambling as more risky than skydiving, while another individual whose risk attitudes are related to physical danger might have the opposite perspective. The current findings also complement previous demonstrations that self-reported attitudes to risk in general predict risk in diverse decisions [17]. The present research extends these findings by formally identifying domain-general and domain-specific aspects of risk attitudes and testing both as predictors of actual economic risk taking. These findings also dovetail with other reports of domain-general psychological processes [18–20] and empirical examinations regarding the relationship between stated and revealed preferences.

These findings are also consistent with a prior demonstration that a domain-general component can be extracted from the DOSPERT [5] and used to predict counterproductivity. However, our work departs from and extends these findings in several important ways. First, this prior work employed a bifactor model to extract a domain-general component to risk attitudes. Instead, here we employed a second-order model to extract the domain-general component, which is the more common approach [21] having been used widely in the fields of personality, self-concept, and psychological well-being [28–30]. Importantly, this approach is better suited when the focus is on identifying whether the correlation between first-order, domain-specific factors is attributable to a higher-order, domain-general factor. Compared to the bifactor model, the second-order model employed here is also more parsimonious [21]. Importantly, the present finding using the second-order model revealing a domain-general component to risk attitudes is consistent with other empirical demonstrations using the bifactor model [3,5], providing evidence of the robustness of this effect. Second, we extend this prior work by examining incentive-compatible economic decision making, which was not investigated in this prior work [3,5]. Critically, we also demonstrate the relative importance of domain-general and domain-specific risk attitudes to economic decision making, revealing that domain-general but not domain-specific risk attitudes shape financial risk taking. Here, domain-general risk attitudes predict incentive-compatible decisions, rather than being derived from them (in contrast to, e.g., [4]). Our findings therefore both complement and extend those from prior research.

Our findings do depart somewhat from a prior demonstration by Dohmen and colleagues [17] that domain-specific measures of risk attitudes better predict behavior within different domains than domain-general risk attitudes. Instead, we demonstrate that domain-general risk attitudes better predict economic risk taking than domain-specific risk attitudes. There may be several reasons for these discrepant findings. For example, whereas our domain-general and domain-specific attitudes were extracted from the same measures using confirmatory factor analysis, their measures were collected via separate, one-item surveys. Additionally, their domain-specific risk behaviors were derived from single self-report measures, whereas our behavior was derived from an incentive-compatible task allowing precise estimation of behavioral parameters. Furthermore, while we focused on financial decision making, their work examined behaviors in other domains, including career and health. Future work should examine the circumstances under which the relative contributions of domain-general and domain-specific risk attitudes may vary, including examining different methods of soliciting behavioral measures and diverse decision-making contexts. Note, though, that our findings complement Dohmen and colleagues’ key conclusion that domain-general attitudes are essential to understanding risk.

One feature of the present analyses is that cross-loadings between items from different factors were freed from the original model, making this analysis less strictly confirmatory. As a supplemental analysis, we tested the original measurement model in the structural model. This model not only exhibited poor fit (χ^2^(772) = 1304.28, p < .001; RMSEA = .048, 90% CI = .043, .053; CFI = .880), but also was a significantly weaker model when directly compared with the proposed initial structural model (χ^2^(9) = 199.75, *p* < .001). Thus the original measurement model is not a good candidate for testing the structural model, and the modified model was retained for primary analyses. Importantly, this approach is related to recent advances in exploratory structural equation modeling [26,27], which allow cross-loadings for items to deviate from zero.

The present findings also complement neuroimaging studies regarding the neural substrates of risky decision making. Meta-analyses and review articles have linked decision making under risk to a core network of regions, including orbitofrontal cortex and anterior cingulate cortex [31,32]. This network may be recruited in a domain-general fashion, consistent with the present finding that domain-general risk attitudes are central to shaping behavior. Future neuroimaging studies may seek to disentangle whether separable domain-specific and domain-general aspects of risky decision making are represented neurally.

Decisions about risk often involve important choices in areas where people have limited knowledge and experience, such as deciding about a medical treatment after receiving a cancer diagnosis. This lack of experience creates challenges in quantifying risk attitudes in order to advise people in these circumstances [33]. The Financial Services Authority highlights this challenge, emphasizing the large number of investment decisions that are inappropriate given consumers’ risk attitudes [34]. The present findings might be used to help promote consumer welfare by suggesting the incorporation of risk attitudes from diverse but familiar domains like health and recreation as guides for consumers’ financial planning and investing.

## Supporting information

S1 Fig Schematic depiction of a trial from the risk preference task. On each trial, participants were presented with a certain option (e.g., guaranteed payment of $5) and a risky option (e.g., a gamble with a 50% chance of winning $16 and a 50% chance of winning nothing). Participants indicated which option they preferred via keyboard button press.
(DOCX)

S1 Table Means, standard deviations, and correlations among observed variables. (PDF)

S2 Table Measurement model maximum likelihood estimates and test of free parameters from DOSPERT responses. (PDF)

S3 Table Structural model maximum likelihood estimates and test of free parameters from DOSPERT responses and risk premium data. (PDF)

S1 Supplemental materials (DOCX)
