# Supplementary material for: Beyond money: Risk preferences across both economic and non-economic contexts predict financial decisions
Source: PLoS One. 2022 Dec 16;17(12):e0279125. doi: 10.1371/journal.pone.0279125 (PMC9757577; doi:10.1371/journal.pone.0279125)
Supplement: S1 Fig — On each trial, participants were presented with a certain option (e.g., guaranteed payment of $5) and a risky option (e.g., a gamble with a 50% chance of winning $16 and a 50% chance of winning nothing). Participants indicated which option they preferred via keyboard button press. (DOCX) [file pone.0279125.s001.docx]

**Supplemental Fig. 1.**

Schematic depiction of a trial from the risk preference task. On each trial, participants were presented with a certain option (e.g., guaranteed payment of $5) and a risky option (e.g., a gamble with a 50% chance of winning $16 and a 50% chance of winning nothing). Participants indicated which option they preferred via keyboard button press.
