# Supplementary material for: Beyond money: Risk preferences across both economic and non-economic contexts predict financial decisions
Source: PLoS One. 2022 Dec 16;17(12):e0279125. doi: 10.1371/journal.pone.0279125 (PMC9757577; doi:10.1371/journal.pone.0279125)
Supplement: S1 Table — (PDF) [file pone.0279125.s002.pdf]

Supplementary Table 1.
Means, standard deviations, and correlations among observed variables.

Item	M	sd	1	2	3	4	5	6	7	8	9	10	11	12	13	14
**1**	4.29	0.78	-													
**2**	3.32	1.39	-.03	-												
**3**	1.50	0.95	-.14	.18	-											
**4**	1.82	1.27	.04	.17	.34	-										
**5**	1.63	0.90	.02	.15	.38	.48	-									
**6**	2.09	1.24	-.04	.42	.32	.27	.34	-								
**7**	3.94	0.96	-.06	.11	.07	-.01	.03	.10	-							
**8**	2.79	1.49	-.04	.18	.30	.49	.26	.16	.02	-						
**9**	1.35	0.74	.02	.07	.35	.51	.43	.30	.12	.40	-					
**10**	3.86	1.07	.32	.04	.08	.17	.23	.11	.12	.10	.19	-				
**11**	1.64	1.09	-.24	.19	.73	.35	.27	.37	.22	.35	.44	.19	-			
**12**	1.67	1.04	.01	.10	.12	.41	.27	.21	.05	.28	.40	.15	.24	-		
**13**	2.19	1.16	.04	.12	.20	.28	.40	.22	.14	.25	.43	.15	.31	.35	-	
**14**	1.42	0.69	-.15	.07	.19	.28	.51	.19	.04	.22	.58	.18	.37	.33	.56	-
**15**	2.44	1.39	-.01	.51	.24	.23	.22	.43	.13	.22	.18	.19	.29	.20	.28	.16
**16**	4.04	0.99	.27	.08	.04	.06	.14	.16	.01	.06	.04	.42	.12	.13	.21	.05
**17**	2.21	1.35	.02	.36	.15	.18	.18	.38	.02	.29	.14	.07	.23	.08	.08	.15
**18**	2.60	1.14	-.16	.27	.36	.18	.14	.26	.27	.22	.39	.13	.48	.26	.26	.23
**19**	3.26	1.09	.22	.11	.02	.17	.12	.13	.19	.07	.25	.22	.11	.11	.20	.06
**20**	3.48	1.33	-.02	.12	.10	.33	.31	.11	.11	.28	.35	.12	.25	.23	.44	.35
**21**	3.19	1.31	-.05	.51	.23	.14	.23	.33	.16	.27	.19	.05	.21	.05	.16	.21
**22**	1.76	1.12	-.23	.12	.67	.18	.28	.25	.22	.30	.38	.35	.72	.28	.34	.37
**23**	3.07	1.11	.19	-.08	-.12	-.18	-.12	-.11	.02	-.15	-.19	.06	-.11	-.05	-.06	-.10
**24**	3.89	1.01	-.03	.13	.00	.03	-.04	.03	.49	.09	.05	.05	.11	.12	.12	.02
**25**	1.60	1.00	-.02	.06	.05	.39	.52	.28	-.02	.27	.35	.18	.28	.47	.51	.52
**26**	3.17	1.33	.26	.08	-.05	.18	.11	-.02	.07	.19	.17	.31	.14	.33	.32	.13
**27**	2.30	1.44	.14	-.01	.05	.32	.24	.07	.04	.32	.25	.17	.14	.34	.29	.26
**28**	2.69	1.41	.03	.17	.13	.32	.34	.15	.15	.35	.37	.14	.25	.27	.45	.28
**29**	1.92	1.25	-.04	.00	.17	.17	.27	.07	-.09	.21	.22	.12	.18	.16	.19	.24
**30**	3.18	1.12	-.06	.05	.09	-.11	-.11	.15	.35	-.11	.05	.03	.15	.07	.01	.06
**31**	3.28	1.37	-.06	.57	.17	.21	.25	.48	.09	.29	.13	.05	.24	.06	.26	.25
**32**	1.59	1.07	-.03	.25	.29	.19	.29	.31	-.01	.23	.21	.09	.35	.24	.13	.30
**33**	1.38	0.90	-.22	.15	.64	.36	.35	.31	.15	.34	.36	.15	.77	.28	.24	.36
**34**	3.95	1.03	.26	.08	-.07	-.03	-.13	-.03	-.04	-.15	-.04	.15	-.10	.03	.13	-.10

Supplementary Table 1 Continued

Item	M	sd	1	2	3	4	5	6	7	8	9	10	11	12	13	14
**35**	3.87	0.95	.29	.11	-.05	.00	-.02	.13	.03	.03	-.01	.24	-.09	-.01	.15	-.08
**36**	3.87	1.17	.00	.05	-.04	.12	.05	.05	-.04	.19	.20	.07	.14	.16	.20	.09
**37**	3.45	1.47	.03	.36	.24	.15	.23	.42	.05	.29	.22	.19	.25	.03	.23	.24
**38**	3.63	1.42	.00	.43	.13	.13	.16	.44	.08	.20	.26	.14	.20	.19	.19	.15
**39**	2.82	1.33	-.11	.33	.06	.23	.19	.33	.10	.29	.22	.09	.27	.32	.33	.25
**40**	3.10	1.40	.02	.00	.05	.10	.04	.01	.06	.27	.27	.19	.22	.23	.20	.20
**Risk Premium**	.51	.60	-.03	-.05	-.04	-.08	-.13	-.10	-.17	-.14	-.08	-.04	-.17	-.10	-.08	.01

Supplementary Table 1 Continued

**Item**	**15**	**16**	**17**	**18**	**19**	**20**	**21**	**22**	**23**	**24**	**25**	**26**	**27**	**28**	**29**	**30**
**16**	.25	-														
**17**	.44	.25	-													
**18**	.37	.11	.21	-												
**19**	.16	.21	.23	.28	-											
**20**	.15	.21	.09	.15	.05	-										
**21**	.41	.23	.50	.26	.09	.26	-									
**22**	.26	.12	.19	.48	.14	.13	.26	-								
**23**	-.07	.10	.00	-.09	.09	-.09	.01	-.07	-							
**24**	.09	.13	.00	.35	.16	.13	.13	.14	.06	-						
**25**	.15	.04	.17	.16	.11	.28	.11	.27	.01	.03	-					
**26**	.21	.26	.09	.17	.36	.25	.06	.02	.10	.11	.25	-				
**27**	.13	.31	.13	.05	.12	.30	.16	.21	-.02	.05	.19	.22	-			
**28**	.26	.13	.11	.22	.11	.55	.23	.21	-.03	.23	.42	.37	.23	-		
**29**	.01	.10	.20	.11	.16	.12	.08	.24	-.04	-.01	.37	.11	.21	.21	-	
**30**	.10	.02	-.01	.24	.00	-.07	.07	.13	.00	.41	-.07	.00	-.02	.01	.01	-
**31**	.51	.24	.51	.21	.14	.20	.66	.28	-.06	.07	.21	.11	.10	.22	.09	.06
**32**	.24	.09	.43	.33	.07	.11	.21	.39	-.05	-.09	.37	.05	.17	.18	.60	.05
**33**	.24	.03	.18	.42	.15	.12	.13	.64	-.02	-.06	.36	.10	.16	.18	.34	-.04
**34**	.15	.15	-.05	-.01	.13	-.03	.06	-.07	.02	.05	-.09	.08	.06	.08	-.03	-.02
**35**	.22	.47	.11	.07	.26	.12	.13	-.01	.12	.05	-.05	.28	.22	.10	.06	.07
**36**	.11	.26	.12	.10	.18	.15	.02	.19	-.03	.10	.22	.13	.28	.14	.25	-.04
**37**	.40	.26	.44	.19	.11	.15	.47	.27	.04	.06	.18	.15	.14	.23	.19	.14
**38**	.42	.19	.35	.22	.13	.08	.44	.17	-.06	.10	.17	.09	.07	.14	.23	.25
**39**	.30	.15	.27	.29	.05	.22	.35	.20	-.13	.07	.33	.07	.24	.13	.35	.03
**40**	.06	.24	.08	.16	.08	.22	-.02	.17	-.07	-.01	.17	.11	.30	.21	.17	-.07
**Risk Premium**	-.10	-.05	-.11	-.15	-.10	-.03	-.09	-.12	.04	-.09	-.15	-.06	-.04	-.07	-.03	-.02

Supplementary Table 1 Continued

Item	31	32	33	34	35	36	37	38	39	40
**31**	-									
**32**	.30	-								
**33**	.29	.58	-							
**34**	.02	-.11	-.07	-						
**35**	.09	.05	-.15	.25	-					
**36**	.10	.21	.04	-.02	.21	-				
**37**	.65	.36	.23	.02	.04	.17	-			
**38**	.50	.31	.22	.12	.14	.12	.55	-		
**39**	.40	.44	.29	-.06	.07	.34	.28	.41	-	
**40**	.00	.22	.16	.00	.15	.30	-.05	.11	.32	-
**Risk Premium**	-.07	-.10	-.21	.01	.00	.03	-.10	-.08	-.19	-.03
