# Supplementary material for: Beyond money: Risk preferences across both economic and non-economic contexts predict financial decisions
Source: PLoS One. 2022 Dec 16;17(12):e0279125. doi: 10.1371/journal.pone.0279125 (PMC9757577; doi:10.1371/journal.pone.0279125)
Supplement: S2 Table — (PDF) [file pone.0279125.s003.pdf]

Supplementary Table 2.
Measurement Model Maximum Likelihood Estimates and Test of Free Parameters from DOSPERT responses.

**Parameter**	**Parameter Estimate**	**Standard Error**	***p* value**	**Standardized Estimate**
Loadings				
Investing on General Risk	.266	.105	.006	.209
Gambling on General Risk	.859	.128	<.001	.608
Recreational on General Risk	.654	.101	<.001	.546
Health/Safety on General Risk	.293	.161	<.001	.767
Ethical on General Risk[1]			<.001	.809
Social on General Risk	.204	.076	<.001	.432
Item 7 on Investing[1]			<.001	.670
Item 18 on Investing	.553	.108	<.001	.370
Item 24 on Investing	1.136	.210	<.001	.761
Item 30 on Investing	.757	.130	<.001	.507
Item 3 on Gambling[1]			<.001	.745
Item 6 on Gambling	.265	.083	.001	.197
Item 11 on Gambling	1.226	.086	<.001	.913
Item 18 on Gambling	.567	.076	<.001	.422
Item 22 on Gambling	1.105	.077	<.001	.823
Item 32 on Gambling	.447	.112	<.001	.333
Item 33 on Gambling	1.142	.090	<.001	.851
Item 2 on Recreational[1]			<.001	.633
Item 6 on Recreational	.828	.101	<.001	.524
Item 15 on Recreational	1.092	.099	<.001	.691
Item 17 on Recreational	.987	.098	<.001	.625
Item 18 on Recreational	.270	.086	.002	.171
Item 21 on Recreational	1.128	.095	<.001	.713
Item 31 on Recreational	1.311	.094	<.001	.829
Item 37 on Recreational	1.140	.101	<.001	.721
Item 38 on Recreational	1.057	.094	<.001	.669
Item 39 on Recreational	.471	.102	<.001	.298
Item 4 on Health/Safety[1]			.050	.202
Item 8 on Health/Safety	3.423	1.825	<.001	.690
Item 27 on Health/Safety	1.955	1.047	<.001	.394
Item 29 on Health/Safety	2.397	1.321	<.001	.483
Item 32 on Health/Safety	1.895	1.100	<.001	.382
Item 36 on Health/Safety	2.025	1.098	<.001	.408
Item 39 on Health/Safety	2.143	1.160	<.001	.432
Item 40 on Health/Safety	2.067	1.120	<.001	.417
Item 4 on Ethical	.702	.151	<.001	.458
Item 5 on Ethical[1]			<.001	.652
Item 9 on Ethical	1.111	.115	<.001	.725

[1] Parameter fixed at 1

Parameter	Parameter Estimate	Standard Error	*p* value	Standardized Estimate
Loadings Continued				
Item 12 on Ethical	.849	.107	<.001	.554
Item 13 on Ethical	1.081	.095	<.001	.705
Item 14 on Ethical	1.046	.104	<.001	.682
Item 20 on Ethical	.787	.109	<.001	.514
Item 25 on Ethical	1.048	.101	<.001	.684
Item 28 on Ethical	.894	.105	<.001	.583
Item 1 on Social[1]			.002	.249
Item 10 on Social	2.344	.784	<.001	.585
Item 16 on Social	2.819	.938	<.001	.703
Item 19 on Social	2.084	.735	<.001	.520
Item 23 on Social	-.063	.333	.848	-.016
Item 26 on Social	2.460	.864	<.001	.614
Item 27 on Social	.918	.432	.002	.229
Item 34 on Social	.652	.338	.020	.163
Item 35 on Social	2.019	.680	<.001	.503
Covarying Uniquenesses				
Item 29 and 32	.339	.065	<.001	.490
Item 28 and 20	.252	.052	<.001	.362
Variances				
General Risk	.279	.055		
Investing	.430	.100	<.001	.956
Gambling	.350	.058	<.001	.630
Recreational	.281	.046	<.001	.702
Health/Safety	.017	.017	<.001	.411
Ethical	.147	.040	<.001	.345
Social	.051	.033	<.001	.813
