# Supplementary material for: Beyond money: Risk preferences across both economic and non-economic contexts predict financial decisions
Source: PLoS One. 2022 Dec 16;17(12):e0279125. doi: 10.1371/journal.pone.0279125 (PMC9757577; doi:10.1371/journal.pone.0279125)
Supplement: S3 Table — (PDF) [file pone.0279125.s004.pdf]

Supplementary Table 3.
Structural Model Maximum Likelihood Estimates and Test of Free Parameters from DOSPERT responses and risk premium data.

Parameter	Parameter Estimate	Standard Error	*p* value	Standardized Estimate
Structural Model Estimates				
General Risk with Risk Premium	-.191	.083	.018	-.168
Investing with Risk Premium	-.109	.064	.088	-.124
Risk Premium Variance	.339	.033	<.001	.948
Loadings				
Investing on General Risk	.271	.106	.006	.210
Gambling on General Risk	.863	.129	<.001	.611
Recreational on General Risk	.657	.102	<.001	.547
Health/Safety on General Risk	.294	.161	<.001	.767
Ethical on General Risk[1]			<.001	.806
Social on General Risk	.205	.076	<.001	.433
Item 7 on Investing[1]			<.001	.679
Item 18 on Investing	.547	.107	<.001	.372
Item 24 on Investing	1.110	.202	<.001	.755
Item 30 on Investing	.741	.127	<.001	.503
Item 3 on Gambling[1]			<.001	.744
Item 6 on Gambling	.265	.083	.001	.197
Item 11 on Gambling	1.228	.086	<.001	.914
Item 18 on Gambling	.567	.077	<.001	.422
Item 22 on Gambling	1.106	.077	<.001	.823
Item 32 on Gambling	.447	.113	<.001	.332
Item 33 on Gambling	1.145	.090	<.001	.852
Item 2 on Recreational[1]			<.001	.632
Item 6 on Recreational	.829	.101	<.001	.524
Item 15 on Recreational	1.093	.099	<.001	.691
Item 17 on Recreational	.989	.098	<.001	.625
Item 18 on Recreational	.269	.086	.002	.170
Item 21 on Recreational	1.128	.095	<.001	.713
Item 31 on Recreational	1.311	.095	<.001	.829
Item 37 on Recreational	1.141	.102	<.001	.722
Item 38 on Recreational	1.058	.094	<.001	.669
Item 39 on Recreational	.468	.102	<.001	.296
Item 4 on Health/Safety[1]			.049	.202
Item 8 on Health/Safety	3.431	1.818	<.001	.691
Item 27 on Health/Safety	1.947	1.037	<.001	.392
Item 29 on Health/Safety	2.393	1.313	<.001	.482
Item 32 on Health/Safety	1.899	1.097	<.001	.383
Item 36 on Health/Safety	2.014	1.086	<.001	.406

[1]Parameter fixed at 1

Parameter	Parameter Estimate	Standard Error	*p* value	Standardized Estimate
Loadings Continued				
Item 39 on Health/Safety	2.165	1.163	<.001	.436
Item 40 on Health/Safety	2.062	1.112	<.001	.416
Item 4 on Ethical	.701	.150	<.001	.458
Item 5 on Ethical[1]			<.001	.654
Item 9 on Ethical	1.109	.115	<.001	.725
Item 12 on Ethical	.849	.107	<.001	.555
Item 13 on Ethical	1.079	.095	<.001	.705
Item 14 on Ethical	1.040	.104	<.001	.680
Item 20 on Ethical	.784	.109	<.001	.513
Item 25 on Ethical	1.049	.101	<.001	.686
Item 28 on Ethical	.892	.105	<.001	.583
Item 1 on Social[1]			.002	.249
Item 10 on Social	2.346	.786	<.001	.584
Item 16 on Social	2.822	.940	<.001	.703
Item 19 on Social	2.094	.740	<.001	.521
Item 23 on Social	-.066	.334	.840	-.016
Item 26 on Social	2.463	.867	<.001	.613
Item 27 on Social	.920	.433	.002	.229
Item 34 on Social	.650	.338	.020	.162
Item 35 on Social	2.017	.681	<.001	.502
Covarying Uniquenesses				
Item 29 and 32	.339	.065	<.001	.490
Item 28 and 20	.253	.052	<.001	.363
Variances				
General Risk	.277	.055		
Investing	.441	.101	<.001	.956
Gambling	.347	.058	<.001	.627
Recreational	.280	.046	<.001	.700
Health/Safety	.017	.017	<.001	.411
Ethical	.150	.040	<.001	.351
Social	.050	.033	<.001	.813
