# Supplementary material for: Beyond money: Risk preferences across both economic and non-economic contexts predict financial decisions
Source: PLoS One. 2022 Dec 16;17(12):e0279125. doi: 10.1371/journal.pone.0279125 (PMC9757577; doi:10.1371/journal.pone.0279125)
Supplement: S1 Supplemental materials — (DOCX) [file pone.0279125.s005.docx]

Beyond money: Risk preferences across both economic and non-economic contexts predict financial decisions

**Supplemental Methods**

*Confirmatory Factor Analysis*

Following the guidance of previous exploratory factor analyses (Weber, Blais, & Betz, 2002), items 7, 18, 24, and 30 served as indicators for the Investing factor; items 3, 11, 22, and 33 served as indicators for the Gambling factor; items 2, 6, 15, 17, 21, 31, 37, and 38 served as indicators for the Recreational factor; items 4, 8, 27, 29, 32, 36, 39, and 40 served as indicators for the Health/Safety factor, items 5, 9, 12, 13, 14, 20, 25, and 28 served as indicators for the Ethical factor; and items 1, 10, 16, 19, 23, 26, 34, and 35 served as indicators for the Social factor. The loading of the first item listed for each factor was fixed at one and the covariances of all uniquenesses were fixed at zero. Thus, the model included 40 observed variables and estimated the 34 loadings of the observed variables onto the six domain-specific factors, the 6 disturbances for the six domain-specific factors, and 40 uniquenesses. The second comparison model included a second-order domain-general latent variable, with the loading from the Ethical domain fixed at one. This comparison model therefore involved the additional estimation of the five loadings of the domain-specific factors on the domain-general factor and the disturbance of the domain-general factor.

Upon determining that neither initial measurement model provided adequate overall fit to the observed data, Lagrange Multiplier (LM) Tests were conducted to explore possible model modifications. First, the covariances between the uniquenesses of items 28 and 20 and items 32 and 29 were freed. Post-hoc inspection of these items revealed that they exhibited conceptual similarity that was not shared with other items, as the first pair (“Stealing an additional TV cable connection off the one you pay for” and “Illegally copying a piece of software”) both corresponded to ethical transgressions involving digital media and the second pair (“Not wearing a helmet when riding a motorcycle” and “Not wearing a seatbelt when being a passenger in the front seat”) both related to vehicular transport. Second, LM tests also suggested freeing the loadings of items on domain-specific factors beyond their original putative domain. The loading of item 4 (“Buying an illegal drug for your own use”) on the Ethical risk domain was freed. Interestingly, this item was originally developed as an index of Ethical risk tolerance and later assigned to the Health/Safety scale based on exploratory factor analysis (Weber et al., 2002), potentially due to its potential for both health risks as well as legal ramifications. The loading of item 27 (“Engaging in unprotected sex”) on Social risk was also freed following the LM test, an adjustment that was conceptually appealing given its involvement of interpersonal relationships. The loading of item 18 (“Investing 5% of your annual income in a very speculative stock”) on the additional factors of Recreational and Gambling risk was freed, as was the loading of item 39 (“Walking home alone at night in a somewhat unsafe area of town”) on Recreational risk. The loadings of items 6 (“Chasing a tornado or hurricane by car to take dramatic photos”) and 32 (“Not wearing a helmet when riding a motorcycle”) were freed on the Gambling domain factor. We examined factor loadings from published exploratory factor analyses (Weber et al., 2002) and found that these cross-loadings are consistent with that work. Note that all measurement model adjustments were implemented prior to and independently from estimation of the structural model.

As noted in the main text, participants also completed additional measures as part of a larger study of individual differences in risk and reward processing. Participants completed all measures in one session and were instructed to read the instructions and surveys carefully. The DOSPERT employed the standard published instructions, and the incentive-compatible risk preference task utilized the instructions included below.

**Supplemental Results**

Although the main structural models reported in the present article provide evidence that domain-general risk attitudes can shape behavior, some might suggest that the original measurement model based on previous exploratory factor analyses of the DOSPERT might provide enhanced model fit compared to the structural model described in the main text. To address this possibility, an additional structural model was conducted using the original measurement model (with the domain-general factor) and adding paths between both the Investing factor and the domain-general factor and the risk premiums. This model not only exhibited poor fit (χ^2^(772) = 1304.28, p < .001; RMSEA = .048, 90% CI = .043, .053; CFI = .880), but also was a significantly weaker model when directly compared with the proposed initial structural model (χ^2^(9) = 199.75, *p* < .001).

**Sample Participant Instructions**

In this task you will choose between two possible options. Each option type will be represented by a schematic circle, as shown below [Image similar to Supplemental Fig. 1]. For the certain option (Supplemental Fig. 1, left option), the amount of money in the center is the amount that you will receive if this trial is selected. For the risky option (Supplemental Fig. 1, right option), “pie” sections show the probability of each option. In this example, there is a 50% chance of $0 and a 50% chance of $16. If this trial is selected, then we play out the gamble to determine how much money you will receive. You will only see pie sections that correspond to 25%, 50%, and 75%. You will be presented with many trials. For each trial, just choose the option which you would prefer to receive. After you have completed all tasks, we will randomly select one trial, resolve the selected option, and add that amount to your collected money. To select your desired option, use the arrow keys on the keyboard. You have as much time as you need on each trial, but try to go quickly!

References

Weber, E. U., Blais, A. R., & Betz, N. E. (2002). A domain-specific risk-attitude scale: Measuring risk perceptions and risk behaviors. *Journal of Behavioral Decision Making, 15*(4), 263-+. doi:10.1002/bdm.414
